# Glycine-β-Muricholic Acid Improves Liver Fibrosis and Gut Barrier Function by Reducing Bile Acid Pool Size and Hydrophobicity in Male *Cyp2c70* Knockout Mice

**DOI:** 10.3390/cells12101371

**Published:** 2023-05-12

**Authors:** Mohammad Nazmul Hasan, Jianglei Chen, Huaiwen Wang, Yanhong Du, Yung Dai Clayton, Lijie Gu, Tiangang Li

**Affiliations:** 1 Harold Hamm Diabetes Center, Department of Physiology, University of Oklahoma Health Sciences Center, Oklahoma City, OK 73104, USA; 2 Laboratory for Molecular Biology and Cytometry Research, University of Oklahoma Health Sciences Center, Oklahoma City, OK 73104, USA

**Keywords:** bile acid, ASBT, CYP7A1, FXR, cholestasis, CYP2C70

## Abstract

*Cyp2c70* knockout mice lack the enzyme that produces muricholic acids and show a “human-like” hydrophobic bile acid pool-induced hepatobiliary injury. In this study, we investigated the potential anti-cholestasis effect of glycine-conjugated β muricholic acid (G-β-MCA) in male *Cyp2c70* KO mice based on its hydrophilic physiochemical property and signaling property as an farnesoid X receptor (FXR) antagonist. Our results showed that G-β-MCA treatment for 5 weeks alleviated ductular reaction and liver fibrosis and improved gut barrier function. Analysis of bile acid metabolism suggested that exogenously administered G-β-MCA was poorly absorbed in the small intestine and mostly deconjugated in the large intestine and converted to taurine-conjugated MCA (T-MCA) in the liver, leading to T-MCA enrichment in the bile and small intestine. These changes decreased the biliary and intestine bile acid hydrophobicity index. Furthermore, G-β-MCA treatment decreased intestine bile acid absorption via unknown mechanisms, resulting in increased fecal bile acid excretion and a reduction in total bile acid pool size. In conclusion, G-β-MCA treatment reduces the bile acid pool size and hydrophobicity and improves liver fibrosis and gut barrier function in *Cyp2c70* KO mice.

## 1. Introduction

Bile acids are synthesized from cholesterol in hepatocytes and act as physiologic detergents to emulsify dietary lipids and signaling molecules to regulate various aspects of physiology in the enterohepatic system [1]. Cholesterol 7α-hydroxylase (CYP7A1) mediates the rate-limiting step in hepatic primary bile acid synthesis [2]. Bile acids are released into the small intestine in response to food intake and re-absorbed in the terminal ileum to be transported back to the liver. In the gut, bacterial enzymes can further modify bile acids via deconjugation, dihydroxylation, and epimerization reactions to convert primary bile acids into secondary bile acids, which are either excreted or reabsorbed to enter the enterohepatic circulation. Therefore, the bile acid pool is a mixture of primary and secondary bile acids with different hydrophobicity and signaling properties. Bile acids serve as endogenous ligands for nuclear receptor farnesoid X receptor (FXR) [3] and Takeda G protein-coupled receptor 5 (TGR5) [4], which play important roles in regulating cholesterol and bile acid homeostasis, nutrient metabolism, immune response, and cell proliferation [1].

Dysregulation of bile acid homeostasis has been causally linked to many human metabolic and inflammatory diseases [1]. Cholestasis is a major form of liver disease caused by impaired bile flow out of the liver, and the resulting intrahepatic bile acid accumulation leads to parenchymal and bile duct injury [5]. Genetic defects in liver transporters and inflammation and autoimmune-mediated destruction of bile ducts are some of the common causes of cholestasis in humans. For most of the past 3 decades, ursodeoxycholic acid (UDCA), a hydrophilic bile acid, has been the only available treatment for several forms of cholestasis in humans [6]. Mechanistic studies suggest that UDCA provides multi-fold hepatobiliary protection by decreasing bile acid pool hydrophobicity, promoting choleresis, and inhibiting inflammation. A few years ago, FXR agonist obeticholic acid (OCA) was approved as a second treatment, either in combination with UDCA or as a monotherapy, for primary biliary cholangitis, which is caused by the autoimmune destruction of intrahepatic small bile ducts [5,7]. OCA has not been approved for treating other forms of cholestasis. A common adverse effect of OCA is pruritus. Currently, therapeutic options are still limited for patients who do not adequately respond to these available treatments. Fibroblast growth factor 19 (FGF19) analogue and inhibitors of the gut bile acid uptake transporter apical sodium-dependent bile acid transporter (ASBT) have been investigated as potential new therapeutics for cholestasis [8,9,10,11,12].

It is known that bile acids are conjugated to either glycine or taurine in humans, while bile acids in mice are almost exclusively conjugated to taurine [1]. Another notable difference in the human and murine bile acid metabolism is the bile acid pool composition. Although the primary bile acid synthesis pathway is well conserved, the majority of chenodeoxycholic acid (CDCA) is subsequently metabolized to hydrophilic muricholic acids by CYP2C70 in mice [13]. As a result, the mouse bile acid pool contains the primary bile acids taurine-conjugated (T-) CA (T-CA) and MCA (T-MCA), while the human bile acid pool contains the primary bile acids glycine-conjugated (G-) CA (G-CA) and G-CDCA, as well as T-CA and T-CDCA. This difference renders the mouse bile acid pool more hydrophilic and, if accumulated in liver due to cholestasis, less toxic than the human bile acid pool. In addition, MCAs have been demonstrated to act as FXR antagonists [14], while the abundant bile acids in humans CA, CDCA, and DCA all act as FXR agonists [1]. Alteration of CA to MCA ratio in mice has been shown to significantly impact both the physiologic detergent function and signaling property of the bile acid pool [15,16], while alteration of CDCA to CA ratio is expected to have minimal impact on bile acid pool hydrophobicity or signaling property in humans. It has also been shown that treating mice with glycine-conjugated βMCA (G-β-MCA), which is not an abundant naturally occurring bile acid in mice, antagonizes intestine FXR signaling and improves metabolic homeostasis in mice [17]. Recently, *Cyp2c70* knockout mice have been generated. The bile acid pool of the *Cyp2c70* KO mice primarily contains hydrophobic CDCA and CA, and these mice developed hydrophobic bile acid-induced hepatobiliary injury [18,19]. Several studies have utilized *Cyp2c70* KO mice as a model to investigate the efficacy of various therapeutic agents against human-like bile acid pool-induced hepatobiliary injury [20,21,22,23]. Being a hydrophilic bile acid, the potential anti-cholestasis property of G-β-MCA has not been tested. Here, we report that a treatment of G-β-MCA can improve liver fibrosis and gut barrier impairment in *Cyp2c70* KO mice. These beneficial effects may be attributed to a reduction in bile acid pool size and hydrophobicity.

## 2. Materials and Methods

### 2.1. Reagents

Aspartate aminotransferase (AST) and alanine aminotransferase (ALT) assay kits were purchased from Pointe Scientific (Canton, MI, USA). A bile acid assay kit was purchased from Diazyme Laboratories (Poway, CA, USA). Glycine-conjugated β muricholic acid (G-β-MCA) was purchased from MedChemExpress Inc. (Monmouth Junction, NJ, USA). F4/80 antibody (Cat #. 70076) was purchased from Cell Signaling Technology (Danvers, MA, USA). CK19 antibody (ab52625) was purchased from Abcam (Waltham, MA, USA). ZO-1 antibody (PA5-28858) was purchased from ThermoFisher Scientific (Grand Island, NY, USA). Fluorescein isothiocyanate–dextran (FITC–dextran, MW:3000–5000) was purchased from Sigma Aldrich (St. Louis, MO, USA).

### 2.2. Mice

The *Cyp2c70* KO mice were generated by CRISPR/Cas-mediated genome engineering by Cyagen Biosciences Inc. (Santa Clara, CA, USA) as described previously [23]. Mice were housed in micro-isolator cages with Biofresh performance bedding (Pelleted cellulose) under a 7 a.m.–7 p.m. light cycle and a 7 p.m.–7 a.m. dark cycle. The standard chow diet was PicoLab Rodent Diet 20 (#5053, LabDiet, St. Louis, MO, USA), which contained ~13% fat calories and ~62% carbohydrate calories. G-β-MCA was mixed with the powered chow diet, which was subsequently mixed with 5% sterile water and pressed into small pellets. An estimated 20 mg/kg/day G-β-MCA intake was achieved based on 5 g/day food intake by a 25 g mouse. Mice were euthanized by isoflurane after a 6 h fast from 9 a.m. to 3 p.m. and tissue and blood samples were collected. To measure gut permeability, mice were first fasted from 9 a.m. to 3 p.m. Blood was collected between 2:30 p.m.–3 p.m. via tail snip for baseline measurement. FITC–dextran was dissolved in sterile PBS (60 mg/mL) and a single dose (600 mg/kg BW) was given via oral gavage at ~3 p.m. Blood was collected again at 1 h after FITC–dextran administration. FITC fluorescence in serum samples was measured with a Tecan M200 PRO plate reader (excitation, 485 nm; emission, 530 nm). The fluorescent values measured in samples collected before FITC–dextran gavage were used as background measurements, and the serum FITC–dextran concentration was calculated based on a standard curve.

### 2.3. Bile Acid Analysis: Total Bile Acid Measurement and LC-MS Method

Bile acids were extracted with 90% ethanol from the liver, whole gallbladder, whole small intestine with content, and dried feces, as previously described [23]. Fresh fecal samples were collected by placing an individual mouse in a jar briefly. Fecal samples were collected at around 9 a.m. on the day of tissue collection and then air-dried and weighed. The fecal samples were then homogenized in 90% ethanol to extract bile acids. The bile acid pool was calculated as the sum of bile acids in the liver, gallbladder, and small intestine. For LC-MS measurement of bile acids, bile acid extracts were dried and resuspended in injection buffer and detected on a Thermo Scientific UltiMate 3000 UHPLC with a Waters Cortecs C18 column (Waters Acquity UPLC HSS T3 1.8 μm, 2.1 × 150 mm, part No. 186003540) and a TSQ Quantis triple quadrupole mass spectrometer, as described previously [23].

### 2.4. Histology and Immunohistochemistry

Paraffin-embedded tissues were sectioned at 5 mm thickness and stained with hematoxylin and eosin (H&E) or Sirius Red (Direct Red 80 solution, Sigma #365548, St. Louis, MO, USA). For immunohistochemistry, paraffin-embedded tissue sections were deparaffinized and rehydrated, and, after antigen retrieval, were blocked in PBS buffer containing 5% BSA and 5% goat serum for 1 h. These tissue sections were incubated in primary antibodies overnight at 4 °C, washed with PBS, and incubated with secondary antibodies in SignalStain^®^ Boost IHC Detection Reagent (Cell Signaling, #8114, Danvers, MA, USA) for 1 h. Signal was visualized with a DAB kit (Cell Signaling, #11724, Danvers, MA, USA). The tissue sections were counterstained with hematoxylin. Images are acquired with an EVOS M5000 imaging system (ThermoFisher Scientific, Grand Island, NY, USA). Images taken with a 10× lens were shown as representative images. Images taken with a 4× lens were used for quantification of the positive stain area with ImageJ software (NIH).

### 2.5. Real-Time PCR

Liver total RNA was purified with Trizol (Sigma-Aldrich, St. Louis, MO, USA). Total RNA (2 mg) was used in a reverse transcription reaction with Oligo dT primer and SuperScript III reverse transcriptase (ThermoFisher Scientific, Grand Island, NY, USA). Real-time PCR was performed with a Bio-Rad CFX384 real-time PCR system and iQ SYBR Green Supermix (Bio-rad, Hercules, CA, USA). Here, 18S was measured as the internal control for normalization. The comparative CT (Ct) method was used to calculate the relative mRNA expression with the average control value set as “1”. The sequences of real-time PCR primers are included in Table 1.

### 2.6. Statistical Analysis

All results were expressed as mean ± SEM. An unpaired *Student’s* t-test was used to calculate the *p* value. A *p* < 0.05 was considered statistically significant.

## 3. Results

### 3.1. G-β-MCA Treatment Attenuates Ductular Reaction and Liver Fibrosis in Cyp2c70 KO Mice

**Figure 1:**
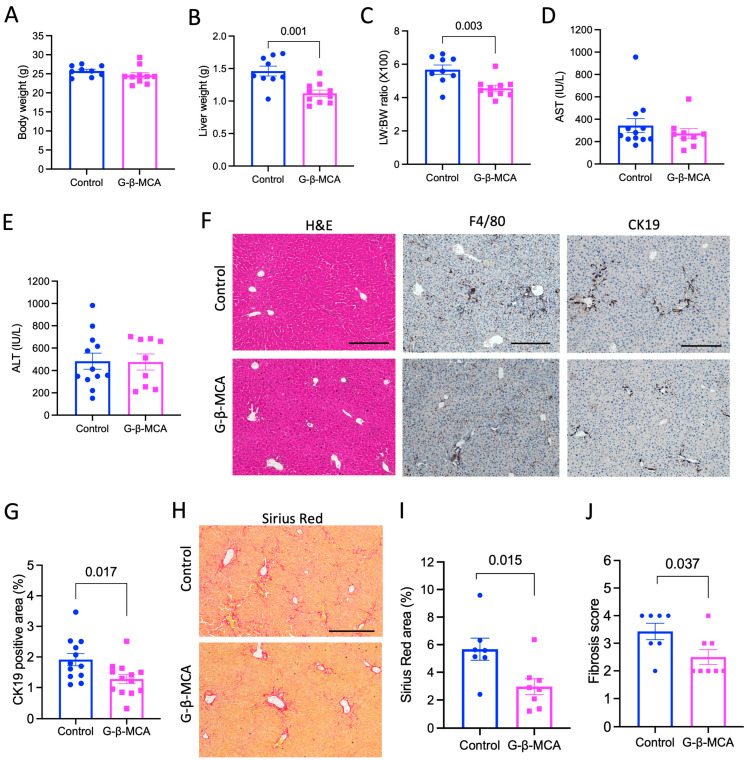
G-β-MCA treatment alleviates liver ductular reaction and fibrosis in *Cyp2c70* KO mice. Male *Cyp2c70* KO mice at 8 weeks of age were fed a G-β-MCA-containing chow diet for 5 weeks. Control mice were age- and sex-matched *Cyp2c70* KO mice fed the same chow diet for 5 weeks. Mice were fasted for 6 h from 9 a.m.–3 p.m. and euthanized. (**A**) Body weight at the end of the treatment. (**B**) Liver weight. (**C**) Liver weight (LW) to body weight (BW) ratio. (**D**,**E**) Serum aspartate aminotransferase (AST) and alanine aminotransferase (ALT) concentration. (**F**) Representative images of H&E stain, immunohistochemistry of F4/80 stain and CK19 stain. Scale bar = 250 mm. (**G**) CK19 positive area was quantified by ImageJ Fiji software. (**H**) Representative Sirius Red stain. Scale bar = 250 mm. (**I**) Sirius Red positive area per view was quantified with ImageJ Fiji Software. (**J**) Ishak fibrosis score. n = 9–13. All results are expressed as mean ± SEM.

**Figure 2:**
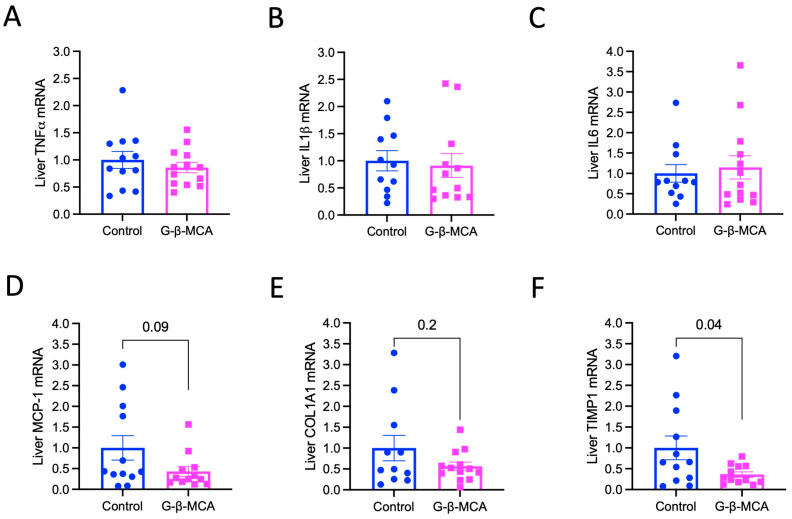
G-β-MCA treatment reduces liver mRNA expression of fibrosis genes but not inflammatory cytokines in *Cyp2c70* KO mice. Male *Cyp2c70* KO mice at 8 weeks of age were fed a G-β-MCA-containing chow diet for 5 weeks. Control mice were age- and sex-matched *Cyp2c70* KO mice fed the same chow diet for 5 weeks. Mice were fasted for 6 h from 9 a.m.–3 p.m. and euthanized. (**A**–**F**). Real-time PCR measurement of liver mRNA expression. Relative liver mRNA expression is expressed with the control set as “1”. n = 11–13. All results are expressed as mean ± SEM.

To investigate the potential anti-cholestasis effect of G-β-MCA, we treated ~8-week-old male *Cyp2c70* KO mice with G-β-MCA for 5 weeks. G-β-MCA treatment did not affect body weight (Figure 1A), but significantly decreased liver weight and liver weight to body weight ratio (Figure 1B,C). A previous study has shown that *Cyp2c70* KO mice showed increased liver weight to body weight ratio than WT mice, possibly due to chronic liver injury [23]. The concentration of the plasma transaminases, namely aspartate aminotransferase (AST) and alanine aminotransferase (ALT), was not significantly altered by the G-β-MCA treatment (Figure 1D,E). Liver histological analysis revealed that *Cyp2c70* KO mice showed portal inflammation (F4/80 stain), ductular reaction (CK-19 stain), and liver fibrosis (Sirius red stain) (Figure 1F,H). We did not observe an apparent reduction in portal inflammatory infiltration upon G-β-MCA treatment. However, we found a significant reduction in CK19 positive stain upon G-β-MCA treatment (Figure 1F,G). In addition, liver fibrosis was significantly attenuated by G-β-MCA treatment (Figure 1H–J). Analysis of gene expression markers showed that G-β-MCA did not lower the liver mRNA of cytokine tumor necrosis factor α (TNFα), interleukin 1β (IL1β), or interleukin 6 (IL6) (Figure 2A–C). The mRNA of chemokine monocyte chemoattractant protein-1 (MCP-1) and fibrosis genes collagen 1A1 (COL1A1) trended lower but did not reach statistical significance, and metallopeptidase inhibitor 1 (TIMP1) was significantly reduced in the G-β-MCA treatment group (Figure 2D–F), which was consistent with the reduction in liver fibrosis in G-β-MCA-treated mice (Figure 1H–J).

### 3.2. G-β-MCA Treatment Improved Gut Barrier Function in Cyp2c70 KO Mice

**Figure 3:**
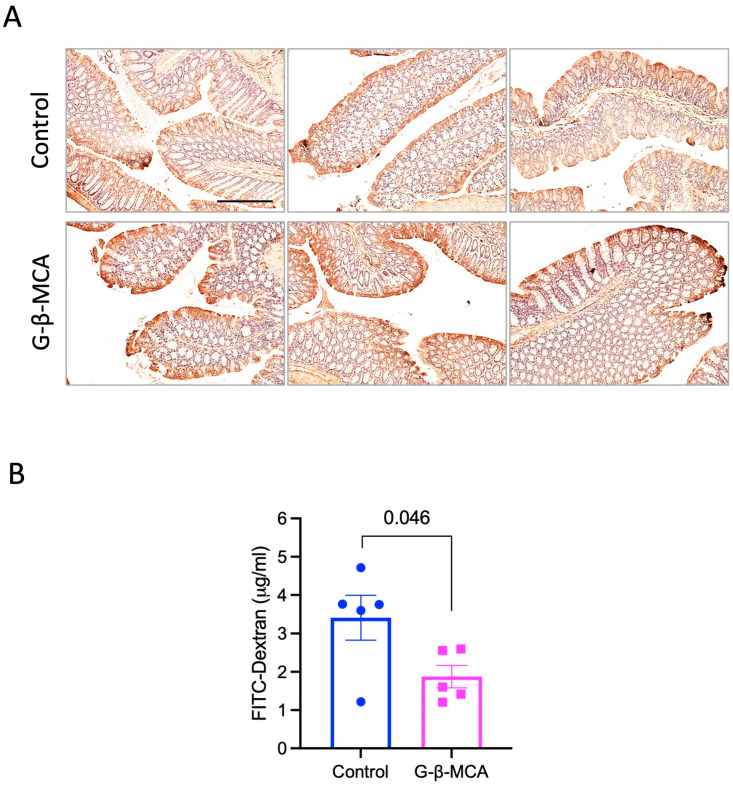
G-β-MCA treatment restores gut barrier integrity in *Cyp2c70* KO mice. (**A**) Male *Cyp2c70* KO mice at 8 weeks of age were fed a G-β-MCA-containing chow diet for 5 weeks. Control mice were age- and sex-matched *Cyp2c70* KO mice fed the same chow diet for 5 weeks. Mice were fasted for 6 h from 9 a.m.–3 p.m. and euthanized. Representative images of colon immunohistochemistry stains of ZO-1 from three mice per group are shown. Scale bar = 250 mm. (**B**) Male *Cyp2c70* KO mice at 8 weeks of age were fed a G-β-MCA-containing chow diet for 5 weeks. Control mice were age- and sex-matched *Cyp2c70* KO mice fed the same chow diet for 5 weeks. Mice were fasted for 6 h from 9 a.m.–3 p.m. and FITC–dextran was administered as described in the Materials and Methods section. Serum FITC–dextran concentration is shown. n = 5. All results are expressed as mean ± SEM.

Impaired gut barrier function is known to contribute to liver inflammation and injury via the gut–liver axis [24]. Studies from us and other have shown that *Cyp2c70* KO mice show impaired gut barrier function due to hydrophobic bile acid exposure [21,23]. Our previous study showed that impaired gut integrity was reflected in a marked reduction in zonula occludens-1 (ZO-1) staining in the colon epithelial cells [24]. Here, we found that mice treated with G-β-MCA appeared to show higher ZO-1 intensity in the colon epithelial cells (Figure 3A), suggesting that G-β-MCA may improve gut barrier integrity in *Cyp2c70* KO mice. To obtain further functional evidence to support this effect, we further tested the effect of G-β-MCA on gut permeability by orally administering FITC–dextran to mice. Indeed, blood FITC–dextran concentration was significantly lower in G-β-MCA-treated *Cyp2c70* KO mice than in controls (Figure 3B), supporting the idea that G-β-MCA treatment improved gut barrier function in *Cyp2c70* KO mice.

### 3.3. G-β-MCA Treatment Reduced Total Bile Acid Pool Size and Biliary Bile Acid Hydrophobicity

**Figure 4:**
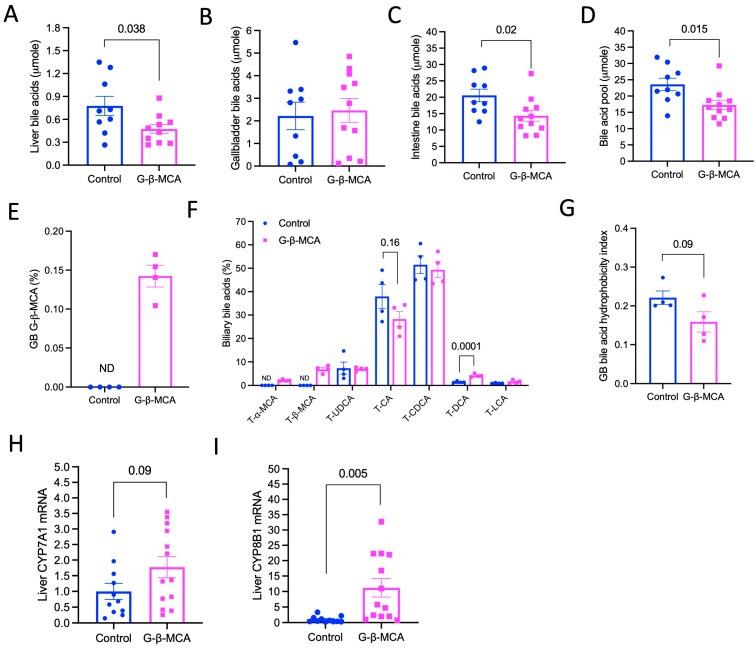
G-β-MCA treatment reduces total bile acid pool and biliary bile acid hydrophobicity in *Cyp2c70* KO mice. Male *Cyp2c70* KO mice at 8 weeks of age were fed G-β-MCA-containing chow diet for 5 weeks. Control mice were age- and sex-matched *Cyp2c70* KO mice fed the same chow diet for 5 weeks. Mice were fasted for 6 h from 9 a.m.–3 p.m. and euthanized. (**A**–**D**) Total bile acid amount in whole liver, gallbladder (GB), small intestine and total bile acids pool. n = 9–11. (**E**,**F**) Relative abundance of individual bile acid in gallbladder bile expressed as percentage of total bile acid concentration in gallbladder bile. n = 4. (**G**) Biliary bile acid hydrophobicity calculated based on bile acid composition shown in “F”. (**H**,**I**) Relative liver mRNA expression is expressed with the control set as “1”. n = 11–13. All results are expressed as mean ± SEM.

To understand how G-β-MCA treatment results in these beneficial effects in *Cyp2c70* KO mice, we next analyzed how G-β-MCA treatment modulated bile acid metabolism. Unexpectedly, despite being orally administered exogenous bile acids, we found that G-β-MCA treatment significantly lowered total liver and small intestine bile acids but not gallbladder total bile acids (Figure 4A–C), which was translated into a ~27% smaller bile acid pool than in untreated controls (Figure 4D). Interestingly, analysis of bile acid composition revealed that G-β-MCA was detectable only in the G-β-MCA-treated mice, but accounted for only less than 0.2% of the total biliary bile acids (Figure 4E). In contrast, the biliary bile of the G-β-MCA-treated mice was enriched with T-αMCA and T-βMCA, which accounted for about ~10% of the total biliary bile acids, while T-αMCA and T-βMCA were not detectable in control mice (Figure 4F). Given that *Cyp2c70* KO mice did not produce endogenous MCA, T-αMCA and T-βMCA detected in the bile of the treated mice were likely derived from the exogenously administered G-β-MCA. G-β-MCA treatment did not alter the relative abundance of T-UDCA, T-CDCA, or T-LCA, but lowered T-CA abundance and increased T-DCA abundance (Figure 4F). As a result of the presence of T-αMCA and T-βMCA, the calculated hydrophobicity index was lower in G-β-MCA-treated mice, although the difference did not reach statistical significance (Figure 4G) [25]. Consistent with the reduced bile acid pool size and biliary bile acid hydrophobicity, G-β-MCA treatment also increased hepatic CYP7A1 and CYP8B1 mRNA expression (Figure 4H,I), suggesting alleviated bile acid repression in these genes.

### 3.4. G-β-MCA Treatment Promotes Fecal Bile Acid Excretion and Reduces Gut Exposure to Hydrophobic Acid

**Figure 5:**
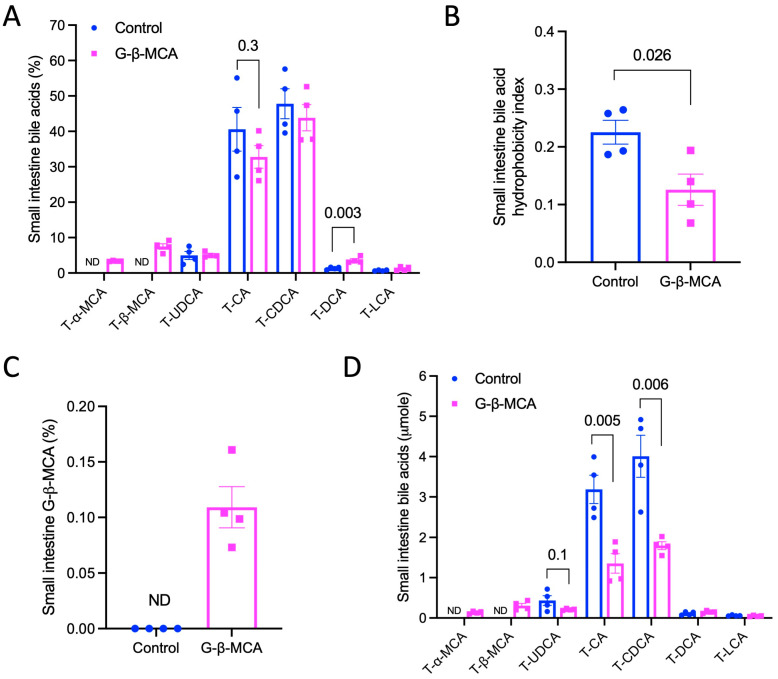
G-β-MCA treatment reduces small intestine bile acid hydrophobicity in *Cyp2c70* KO mice. Male *Cyp2c70* KO mice at 8 weeks of age were fed a G-β-MCA-containing chow diet for 5 weeks. Control mice were age- and sex-matched *Cyp2c70* KO mice fed the same chow diet for 5 weeks. Mice were fasted for 6 h from 9 a.m.–3 p.m. and euthanized. (**A**) Relative abundance of individual bile acid in the small intestine expressed as a percentage of total small intestine bile acids. (**B**) Biliary bile acid hydrophobicity calculated based on bile acid composition shown in (**A**). (**C**) Relative abundance of G-β-MCA in small intestine expressed as percentage of total small intestine bile acids. (**D**) Relative abundance of individual bile acid in the small intestine expressed as absolute amount. n = 4. All results are expressed as mean ± SEM.

It was previously reported that G-β-MCA was resistant to gut bacterial bile salt hydrolase activity and, once orally administered, primarily accumulated in the intestine to elicit its effect via FXR antagonism [17]. To gain a better understanding of how G-β-MCA treatment modulates bile acid metabolism, we next analyzed small intestine bile acid composition. We found that the bile acid composition in the small intestine was similar to that of biliary bile acids, with T-αMCA and T-βMCA accounting for ~10% of total bile acids, a slightly lower T-CA abundance, and a significantly higher T-DCA abundance in the G-β-MCA-treated mice (Figure 5A), leading to a significantly reduced bile acid hydrophobicity index (Figure 5B). Unexpectedly, G-β-MCA was only detected in the treated mice, but accounted for ~0.1% of total bile acids in the small intestine (Figure 5C). As such, the amount of T-αMCA and T-βMCA is ~100-fold higher than G-β-MCA in the small intestine of the treated *Cyp2c70* KO mice. Unconjugated bile acids were largely undetectable in the small intestine of either group, suggesting that bacterial enzyme-mediated bile acid deconjugation primarily occurred in the large intestine. The absolute amount of T-CA and T-CDCA was reduced in the G-β-MCA-treated mice (Figure 5D), which accounted for the significant reduction in total bile acids in the small intestine in these mice (Figure 4C).

**Figure 6:**
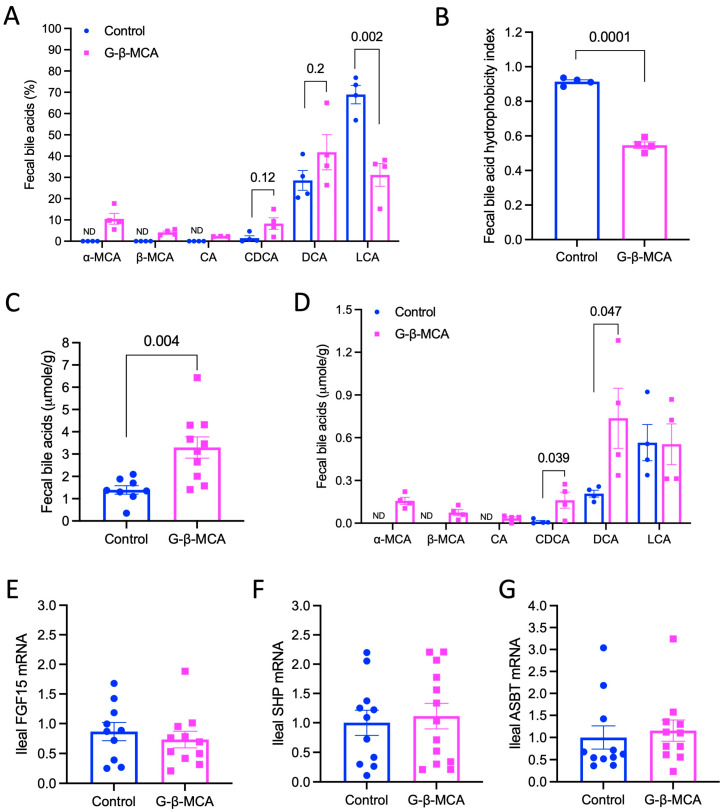
G-β-MCA treatment increases fecal bile acid excretion and decreases fecal bile acid hydrophobicity in *Cyp2c70* KO mice. Male *Cyp2c70* KO mice at 8 weeks of age were fed a G-β-MCA-containing chow diet for 5 weeks. Control mice were age- and sex-matched *Cyp2c70* KO mice fed the same chow diet for 5 weeks. Fresh feces were collected for bile acid analysis. (**A**) Relative abundance of individual bile acid fecal samples expressed as a percentage of total fecal bile acids. n = 4. (**B**) Fecal bile acid hydrophobicity calculated based on bile acid composition shown in (**A**). (**C**) Total fecal bile acids. n = 8–10. (**D**) Relative abundance of individual bile acid in fecal samples expressed as absolute amount. n = 4. (**E**–**G**) Total RNA was extracted from ~1 cm long terminal ileum for mRNA measurement. n = 10–13. All results are expressed as mean ± SEM.

Because G-β-MCA treatment improved gut barrier function (Figure 3), we next analyzed fecal bile acids, which have been shown to closely reflect bile acid composition in the large intestine of *Cyp2c70* KO mice [18]. Fecal bile acids are almost exclusively in the unconjugated forms, and conjugated bile acids, including exogenously administered G-β-MCA, were essentially undetectable. Generally consistent with our previous published results [23], the fecal bile acids of *Cyp2c70* KO controls mice consisted predominantly of highly hydrophobic LCA (~70%) and, to a lesser extent, DCA (~30%), and only trace amount of other bile acids (Figure 6A). While αMCA and βMCA were absent in *Cyp2c70* KO controls as expected, the total amount of αMCA and βMCA accounted for ~15% of total fecal bile acids in the G-β-MCA-treated mice (Figure 6A). Furthermore, LCA abundance decreased to ~30% while DCA and CDCA abundance were modestly higher in the G-β-MCA-treated mice (Figure 6A). Because LCA has the highest hydrophobicity index among all measured bile acids [25], a significant reduction in LCA abundance correlated with a significantly lower fecal bile acid hydrophobicity index in the G-β-MCA-treated mice (Figure 6B).

Furthermore, the G-β-MCA-treated mice showed a ~2-fold higher fecal bile acid content, suggesting that G-β-MCA-treated mice had increased fecal bile acid excretion (Figure 6C), which may explain the reduced small intestine bile acid content and the total bile acid pool in these mice (Figure 4C,D). G-β-MCA treatment did not alter the absolute amount of LCA but significantly increased fecal CDCA and DCA, which, together with αMCA and βMCA, accounted for the overall increased fecal bile acid excretion (Figure 6D). Gene expression analysis found that G-β-MCA treatment did not alter ileal mRNA expression of FXR target gene fibroblast growth factor 15 (FGF15) or small heterodimer partner (SHP) (Figure 6E,F). G-β-MCA treatment did not alter the mRNA of ileal bile acid uptake transporter ASBT (Figure 6G), suggesting that increased fecal bile acid excretion was not a result of downregulation of ileal ASBT expression. In summary, these results show that G-β-MCA treatment promotes fecal bile acid excretion and reduces the gut bile acid pool hydrophobicity.

## 4. Discussion

*Cyp2c70* KO mice show a “human-like” hydrophobic bile acid pool and have become a useful model for the study of bile acid-induced injury in cholestasis. In this study, we investigated the potentially beneficial effect of G-β-MCA treatment in *Cyp2c70* KO mice based on its hydrophilic physiochemical property and signaling property as an FXR antagonist. We have found that G-β-MCA treatment reduced the total and hepatic bile acid pool size and biliary bile acid hydrophobicity. These changes did not improve markers of liver inflammation but attenuated ductular reaction and liver fibrosis. In addition, G-β-MCA treatment improved gut barrier function, which may be attributed to reduced gut bile acid hydrophobicity despite increased fecal bile acid excretion. The absence of endogenous MCAs in *Cyp2c70* KO mice allowed us to obtain a better understanding of how G-β-MCA treatment modulated bile acid metabolism to account for the observed therapeutic benefits, which is further discussed below.

G-β-MCA was previously shown to be poorly absorbed in the small intestine and, therefore, acted as a gut-restricted FXR inhibitor [17]. In addition, it was shown that G-β-MCA was more resistant to hydrolysis by bacterial bile salt hydrolase when compared to the mouse endogenous T-β-MCA [17]. Unexpectedly, our study detected very low levels of G-β-MCA not only in bile but also in the small intestine and feces of the G-β-MCA-treated *Cyp2c70* KO mice. Instead, T-αMCA and T-βMCA were detected in the bile and small intestine and αMCA and βMCA were detected in the feces of the G-β-MCA-treated *Cyp2c70* KO mice. If G-β-MCA can be efficiently absorbed in the terminal ileum like other endogenous bile acids, a higher amount of G-β-MCA than T-MCAs is expected to be present in the bile of the G-β-MCA-treated mice. However, we found that the total biliary concentration of T-αMCA and T-βMCA was about 60 times higher than the biliary G-β-MCA concentration. A plausible explanation for our observation was that G-β-MCA was poorly absorbed in the ileum and most G-β-MCA reached the large intestine where it was deconjugated to βMCA, some of which was further epimerized by bacterial enzymes to αMCA. Both αMCA and βMCA can be passively absorbed in the large intestine and transported to the liver where they are conjugated to become T-αMCA and T-βMCA. In the previous study G-β-MCA hydrolysis by bacterial bile salt hydrolase was tested in fecal protein solution in a 20 min in vitro reaction, and only T-β-MCA, but not G-β-MCA, was found to be rapidly deconjugated [17]. Similarly, our recent study also showed rapid deconjugation of T-CDCA in mouse fecal slurry in vitro [23]. These data suggest that bacterial bile salt hydrolase may show slower kinetics toward G-β-MCA than taurine-conjugated bile acids. Nevertheless, our data suggest the possibility that G-β-MCA can still be efficiently deconjugated during colonic transit under in vivo conditions. The small intestine contained predominantly conjugated bile acids and essentially undetectable unconjugated bile acids, suggesting that quantitatively significant deconjugation of G-β-MCA, after oral intake, only occurred after G-β-MCA reached the large intestine. However, we detected an extremely low amount of G-β-MCA in the small intestine. This may be because we subjected mice to a 6 h fast before tissues were collected. Since the entire gastrointestinal transit time and small intestine transit time in mice were reported to be about 6–8 h and about 2–3 h, respectively [26,27], a 6 h fast was sufficient for most orally acquired G-β-MCA to enter the large intestine to be deconjugated in our study, which provides a possible explanation for the low G-β-MCA level detected in the small intestine of the G-β-MCA-treated mice. As such, orally administered G-β-MCA acts as FXR antagonist in the small intestine and is subsequently converted to T-MCAs to reduce the biliary bile acid pool hydrophobicity.

In our study, the daily G-β-MCA intake per mouse was ~1 μmole/day compared to the total endogenous bile acid pool of 15–20 μmole in *Cyp2c70* KO mice [23]. If the exogenous G-β-MCA is efficiently preserved in the enterohepatic circulation, G-β-MCA treatment over 5 weeks is expected to significantly expand the total bile acid pool in *Cyp2c70* KO mice. On the contrary, G-β-MCA treatment significantly reduced the total bile acid pool of *Cyp2c70* KO mice, which may be attributed to significantly increased fecal bile acid excretion. Interestingly, in addition to fecal αMCA and βMCA excretion, we found that the G-β-MCA-treated mice showed significantly increased fecal excretion of DCA and CDCA, but not LCA. Consistently, we found that T-CA and T-CDCA in the small intestine were significantly lower in the G-β-MCA-treated mice. Efficient conversion of T-CA to DCA in the large intestine can explain why DCA and, to much less extent, CA were enriched in the feces of the G-β-MCA-treated mice. In contrast, T-CDCA did not drive a further increase in fecal LCA enrichment, and, therefore, fecal CDCA was significantly increased in the G-β-MCA-treated mice. These changes suggested that intestine bile acid absorption was reduced in the G-β-MCA-treated mice. In contrast, reduced liver bile acids and unaltered gallbladder bile acids did not suggest that reduced biliary bile acid secretion accounted for lower small intestine bile acids. G-β-MCA treatment did not reduce ileal ASBT expression. Whether G-β-MCA may competitively inhibit endogenous bile acid uptake by ileal ASBT requires investigation. Furthermore, it remains to be determined if reduced ileal bile acid absorption by G-β-MCA treatment is a direct action of G-β-MCA on the bile acid transporter or if it is indirectly caused by altered bile acid composition.

In this study, we found that reduced bile acid pool size and hydrophobicity correlated with modestly elevated hepatic CYP7A1 mRNA expression and a more robust induction of hepatic CYP8B1 mRNA, which was consistent with bile acid feedback inhibition of bile acid synthesis genes [1]. Increased hepatic CYP8B1 could explain the increased production of T-CA, which was subsequently converted to T-DCA in the G-β-MCA-treated mice. We have also reported liver CYP8B1 induction and increased T-DCA abundance by ASBT inhibitor treatment in *Cyp2c70* KO mice [23], suggesting that these changes may be a common response to blocked intestine bile acid uptake. However, we did not see reduced ileal SHP or FGF15 expression in the G-β-MCA-treated mice, which was unexpected, since the total bile acid amount was reduced and FXR antagonists T-MCAs were enriched in the small intestine of these mice. It should be noted that bile acid signaling in the enterohepatic circulation may be significantly altered at baseline in *Cyp2c70* KO mice compared to WT mice due, at least in part, to altered bile acid composition and the presence of inflammation and injury. These changes may underlie differential responses to stimuli in *Cyp2c70* KO mice and WT mice.

In summary, we have shown that G-β-MCA treatment reduces bile acid pool size and hydrophobicity, which contributes to alleviated liver fibrosis and improved gut barrier function in *Cyp2c70* KO mice. However, it should be noted that G-β-MCA treatment did not cause a robust reduction in hepatic inflammation and injury markers, which could be because biliary MCA enrichment was relatively modest. In this study, we chose a ~20 mg/kg G-β-MCA daily dose, which was within the previously tested dosing range in obese mice [17]. Future studies may test if a higher dose of G-β-MCA can provide a more robust improvement in liver pathology in *Cyp2c70* KO mice. The anti-cholestasis effect of G-β-MCA remains to be tested in other cholestasis mouse models with high endogenous levels of MCAs. However, it should be considered that other mouse models contain significant amounts of T-MCAs, which could mask some of the therapeutic benefits of exogenously administered G-β-MCA. Given that the human bile acid pool is enriched with glycine-conjugated bile acids, it can be reasonably speculated that G-β-MCA can be efficiently deconjugated by the gut microbiome in humans. However, it is unclear if G-β-MCA can be efficiently absorbed in human ileum, which may be a key determinant of the extent of G-β-MCA enrichment in the enterohepatic circulation and subsequent reduction in biliary bile acid hydrophobicity. Currently, UDCA remains the first line treatment of many forms of cholestasis in humans [6]. Mechanistic studies suggest that UDCA reduces bile acid pool hydrophobicity, promote biliary HCO_3_^−^ secretion and reducing circulating bile acids and pruritus [6]. However, high doses of UDCA may also increase LCA production, contributing to treatment-associated adverse events [28]. It remains to be tested if G-β-MCA, and possibly other MCA species, shares similar mechanisms of actions with UDCA, and if G-β-MCA can provide beneficial effects in treating certain forms of human cholestasis.

## Figures and Tables

**Table 1:** Mouse real-time PCR primers.

Gene Name	Forward Primer	Reverse Primer
18S	GAGCGAAAGCATTTGCCAAG	GGCATCGTTTATGGTCGGAA
COL1A1	GCTCCTCTTAGGGGCCACT	CCACGTCTCACCATTGGGG
TIMP1	GCAACTCGGACCTGGTCATAA	CGGCCCGTGATGAGAAACT
SHP	TGGGTCCCAAGGAGTATGC	GCTCCAAGACTTCACACAGTG
MCP1	TTAAAAACCTGGATCGGAACCAA	GCATTAGCTTCAGATTTACGGGT
TNFA	CCCTCACACTCAGATCATCTTCT	GCTACGACGTGGGCTACAG
IL1B	GCAACTGTTCCTGAACTCAACT	ATCTTTTGGGGTCCGTCAACT
IL6	TAGTCCTTCCTACCCCAATTTCC	TTGGTCCTTAGCCACTCCTTC
CYP7A1	GGGATTGCTGTGGTAGTGAGC	GGTATGGAATCAACCCGTTGTC
CYP8B1	CCTCTGGACAAGGGTTTTGTG	GCACCGTGAAGACATCCCC
ASBT	GTCTGTCCCCCAAATGCAACT	CACCCCATAGAAAACATCACCA
FGF15	ATGGCGAGAAAGTGGAACGG	CTGACACAGACTGGGATTGCT

## Data Availability

All reported data are contained within this article. Raw data are available upon request.

## Abbreviations

FXR, farnesoid X receptor; CYP7A1, cholesterol 7α-hydroxylase; FGF15, fibroblast growth factor 15; FGF19, fibroblast growth factor 19; UDCA, ursodeoxycholic acid; OCA, obeticholic acid; ASBT, apical sodium-dependent bile acid transporter; KO, knockout; MCA, muricholic acid; CDCA, chenodeoxycholic acid; T-CDCA, tauro-conjugated CDCA; T-CA, tauro-conjugated cholic acid; UDCA, ursodeoxycholic acid; T-LCA, tauro-lithocholic acid; T-DCA, tauro-deoxycholic acid; CYP8B1, sterol 12α-hydroxylase; CK19, cytokeratin-19; AST, aspartate aminotransferase; ALT, alanine aminotransferase; COL1A1, collagen, type I, α1; SHP, small heterodimer partner; ZO-1, zonula occludens-1; TIMP1, metallopeptidase inhibitor 1; G-β-MCA, glycine-conjugated β muricholic acid; TGR5, Takeda G protein-coupled receptor 5; TNFα, tumor necrosis factor α; IL1β, interleukin 1β; IL6, interleukin 6; FITC–dextran, fluorescein isothiocyanate-conjugated–dextran.
